# Determinants and outcomes associated with decisions to deny intensive care unit admission in Tunisian ICU

**DOI:** 10.11604/pamj.2018.29.176.13099

**Published:** 2018-03-26

**Authors:** Rania Bouneb, Menel Mellouli, Maha Dardouri, Houda Ben Soltane, Imed Chouchene, Mohamed Boussarsar

**Affiliations:** 1 Department of Intensive Care Unit, University Hospital of Farhat Hached, Susah Tunisia; 2 Department of Preventive Medicine, Faculty of Medicine, Susah Tunisia; 3 Department of emergency, University Hospital of Farhat Hached, Susah Tunisia

**Keywords:** Intensive care unit, mortality, risk factors

## Abstract

**Introduction:**

intensive care unit (ICU) beds are a scarce resource, and admissions may require prioritization when demand exceeds supply. However, there are few data regarding both outcomes of admitted patients to intensive care unit (ICU) in comparison with outcomes of not admitted patients. The aim of this study was to assess reasons and factors associated to refusal of admission to ICU as well as the impact on mortality at 28 days and patients' outcomes.

**Methods:**

Single-center, cross-sectional descriptive study conducted in 8-bed Medical ICU at a Tunisian University hospital. All consecutive adult patients referred for admission to ICU during 6 months were included. We collected demographic data, ICU admission/refusal reasons, co-morbidity and diagnosis at time of admission, mortality probability model (MPMII0) score, day and time of admission, request for admission and mortality at 28 days.

**Results:**

327 patients were evaluated for ICU admission and 260 were refused to ICU (79.5%). Patients refused because of unavailability of beds represented 50% and patients considered “too sick to benefit” represented 22%. Multivariate analysis showed that the presence of acute respiratory failure and request by direct contact in the unit were independently associated to admission to ICU (OR: 0.15; 95% CI: 0.07-0.31 and OR: 0.16; 95% CI: 0.08-0.31, respectively). Higher mortality rates were shown in patients “too sick to benefit” (80.7%) and unavailable beds (26.56%).

**Conclusion:**

Refusal of ICU admission was correlated with the severity of acute illness, lack of ICU beds and reasons for admission request. ICU clinicians should evaluate their triage decisions and, if possible, routinely solicit patient preferences during medical emergencies, taking steps to ensure that ICU admission decisions are in line with the goals of the patient. Ultimately, these efforts will help ensure that scarce ICU resources are used most effectively and efficiently.

## Introduction

A resuscitator's first goal is to prevent unnecessary suffering and premature death by taking care of the reversible pathologies present for an appropriate period of time. This must be associated with a benefit in terms of both morbidity and mortality, compared to the results obtained by traditional hospital care [1, 2]. However, the admission of patients to intensive care unit (ICU) could be delayed or refused for various reasons. Thus, it is important to evaluate the indications of treatment and their consequences when deciding to admit patients to intensive care [3, 4]. According to the literature, several factors could justify the refusal of patients' admission to ICU. The most common reasons is the health status of the patients: too sick and too well to benefit [5-8]. Moreover, the severity of illness, diagnosis group, nonsurgical status, full unit or unavailable beds in the unit, older age, refusal of the patient/family to be admitted as well as phone admission and daytime admission predict refusal of admission to ICU [1, 5, 7, 9-11]. On the other hand, the refusal of admission of patients could be considered in some situations as a decision to limit or stop therapy [12]. Consequently, studies demonstrated that mortality rates were increased among refused patients and those who were too sick to benefit from resuscitation [8, 11, 13]. While several studies have provided information on the reasons and/or predictors of ICU admission and refusal, few studies have evaluated both outcomes of admitted patients in comparison with outcomes of not admitted patients, and the impact of ICU refusal on mortality. To provide stronger evidence on predictors of ICU refusal, impact on mortality and patients' outcomes, we performed a single center study in a Tunisian university hospital. Our study evaluated reasons and factors associated to refusal of admission to ICU as well as the impact on mortality at 28 days and patients' outcomes.

## Methods

**Population and study design:** This is a single-center, cross-sectional descriptive study conducted in a 8-bed Medical ICU at the University Hospital Farhat Hached in Sousse (Tunisia) over a period of 6 months (from 1 January 2016 to 31 June 2016). In this period, the hospital served a total of 680 beds. It should also be noted that there is a surgical resuscitation of 6 beds for patients requiring medical and surgical management in the same hospital. There is also a 12-bed ICU for obstetric-gynecological and post-operative ICU in some departments such as Otolaryngology (Ear, Nose and Throat) and General Surgery. All consecutive adult patients referred for admission to ICU, during the period of the study were included. The population was divided into two groups: The first is a control group including patients admitted to ICU during the study period, and the second one comprises all the patients who were subject to refusal of admission to the medical ICU. Decisions regarding admission were made by senior ICU physicians, who followed usual admission criteria, without predefined protocol.

**Data collection:** The following data were collected for all patients referred to the ICU: age, gender, co-morbidity, diagnosis at time of admission, mortality probability model (MPMII0) score, day and time of admission, request for admission (by phone; direct contact). Data for scoring MPMII0 were completed by the senior ICU physician attending the case at the time of admission. Reasons for refusal of ICU admission were recorded using multiple-choice items which included patient “too sick to benefit”; patient “too well to benefit”; unavailable beds; collegial decision of therapy arrest; inappropriate referral sites; others (e.g. patient/family refusal ICU of admission). Outcomes evaluated were mortality at 28 days after the day of admission. The study was approved by the research ethics committee of Farhat Hached University Hospital Center of Sousse. Informed and voluntary consent, oral and written, was obtained from all patients participated in the study. All the data obtained were considered as confidential and anonymous.

**Statistical analysis:** Descriptive statistics were used for description of the sample. The categorical variables were described as frequency and percentage and the quantitative variables were described as mean, standard deviation. To compare categorical variables we used the chi-square test. For the comparison of a categorical variable to a quantitative variable, t student test was used. Logistic regression models were built for multivariate analysis to identify factors associated to refusal of admission to ICU. Univariate analysis of main variables registered at referral time was done. Predictor variables that were statistically significant (p < 0.05) were included in logistic regression analysis. The results were presented as odds ratios with the appropriate 95% confidence interval. In logistic regression model, a value of p < 0.2 was considered as independently associated to ICU refusal. The analysis was performed using the statistical package of social science (SPSS 18.0) for Windows.

## Results

**Figure 1:**
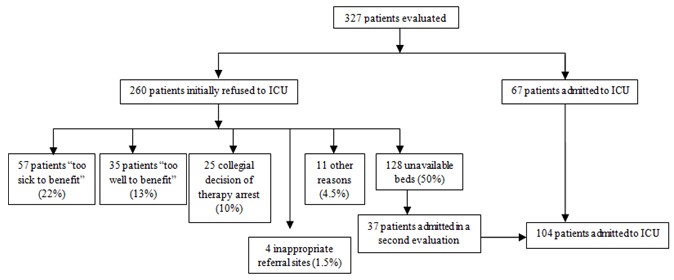
Flow chart of inclusion and exclusion of patients with reasons for ICU refusal

During the study period 327 patients were evaluated for ICU admission, among which 67 patients were admitted (Figure 1). The refusal rate was 79.5% and the main reasons for refusing admission were unavailability of beds (50%) and patients considered “too sick to benefit” (22%). It is important to note that among 260 patients, only one patient refused the transfer to ICU and 3 cases of refusal were by the family of the patient. The request came from different sites of the same hospital: the emergency medical service (3.7%), emergency room (30%), other services (35.7%); also from other hospitals (30.6%).

**Factors associated to refusal of ICU admission:** The baseline characteristics of all patients and the associated factors to refusal of ICU admission are shown in Table 1. There were statistical significant differences between the group of accepted versus refused patients in the following factors: day of admission, time of admission, request for admission and the absence of acute respiratory failure diagnosis (p < 10-3 in all comparisons). Although the mean age of admitted patients was higher than this of refused patients, there was no significant difference between the two parameters (p = 0.55). On the other hand, the mean of MPMII0 of refused patients was higher than which of admitted patients. However, there was no significant difference between MPMII0 and refusal of ICU admission (p = 0.15). Despite refused patients with co-morbidity were more than admitted patients with co-morbidity, no significant difference was found between the presence of co-morbidity and ICU refusal (p = 0.25). Table 2 showed the logistic regression analysis results. The presence of acute respiratory failure and request by direct contact in the unit were independently associated to admission to ICU (OR: 0.15; 95% CI: 0.07-0.31 and OR: 0.16; 95% CI: 0.08-0.31, respectively). Indeed, the absence of acute respiratory failure and the request by phone were predictive factors of refusal of ICU admission.

**Table 1:** Univariate analysis of factors associated to refusal of ICU admission including the characteristics of the participants

Characteristics of patients	Not admitted (n=260)	Admitted (n=67)	*P*
**Age** (years) (m±SD)	55±19	56±18	0.55
**Gender- ratio** (M/F)	170/90	43/24	0.48
**Co-morbidity**, n(%)	222(85.4)	60(89.6)	0.25
**Patient totally dependent**, n(%)	21(8.1)	4(6)	0.39
**MPMII0** (m±SD)	30±25.55	26±20.28	0.15
**Day of admission**, n(%)			
**Holiday and weekend**	260(100)	17(25.4)	p<10^-3^
**Ordinary day**	0(0)	50(74.6)	
**Time of admission**, n(%)			
8 a.m.-6 p.m.	164(65.6)	55(82)	p<10^-3^
6 p.m.-8 a.m.	86(34.4)	12(18)	
**Request for admission**, n(%)			
By phone	161(61.9)	17(25.4)	p<10^-3^
By direct contact	99(38.1)	50(74.6)	
**Diagnosis at time of admission**, n(%)			
**Acute respiratory failure**	137(47.3)	57(85.1)	p<10^-3^
**Acute circulatory failure**	54(20.8)	12(17.9)	0.37
**Heart failure**	17(6.5)	3(4.5)	0.38
**Coma / consciousness disorders**	61(23.5)	9(13.4)	0.23
**Visceral failure**	8(3.1)	7(10.4)	0.18
**Metabolic factors**	12(4.6)	1(1.5)	0.29

**Table 2:** Logistic regression analysis to identify factors independently associated to ICU admission

**Factors**	***p***	**OR**	**IC 95%**
**MPMII_0_**	0,41	1,005	
**Diagnosis at time of admission**			[0.07-0.31]
**Acute respiratory failure**	10^-3^	0.15	
**Visceral failure**	1	0.75	
**Neurological factors**	0.09	0.07	
**Request for admission –By direct contact**	10^-3^	0.16	[0.08-0.31]
**Day of admission**	0.99	0.00	
**Time of admission**	0.99	0.00	

**Outcomes of patients refused to ICU admission and impact on mortality:** Eighty-four (32.3%) of the refused subjects were admitted in a second evaluation: 37 patients in our ICU and 47 in other ICU in the same town. Only one patient among the subjects readmitted in the second evaluation presented an improved health status and he was transferred to another service. However, among the subjects not readmitted in the second evaluation, 108 (61.3%) had no improvements and only 68 patients improved (52 outgoing and 16 transferred to other services). Overall, the improvement rate was 25.7%. The impact on mortality among patients who were refused to ICU admission is shown in Table 3. The mortality rate at 28 days was 43.46% of patients not admitted to ICU. Table 4 showed mortality rate in terms of reasons for refusal of ICU admission. Higher mortality rates were shown in patients “too sick to benefit” (80.7%) and unavailable beds (26.56%).

**Table 3:** Effect of refusal of ICU admission on mortality at 28 days after the day of admission

**Mortality**	**Patients not admitted (n=260)**
After 48 hours, n(%)	60(23.07)
After 2-7 days, n(%)	41(15.79)
After 7-28 days, n(%)	12(4.61)
Mortality rate at 28 days, n(%)	113(43.46)

**Table 4:** Mortality in relation to reasons for refusal of admission to ICU

**Reasons for refusal**	**After 48 hours**	**After 7 days**	**After 28 days**	**Mortality rate**
Unavailable beds (n=128), n (%)	12 (9.37)	17(13.28)	5(3.90)	34(26.56)
Patients « too sick to benefit » (n= 57), n (%)	30 (52.93)	14(22.22)	2(5.55)	46(80.70)
Patients « too well to benefit » (n=35), n (%)	0(0)	1(2.85)	1(2.85)	2(5.71)
Collegial decision of therapy arrest (n=25), n (%)	12(48)	8(32)	2(8)	22(88)
Inappropriate referrals (n=4), n (%)	2(66,66)	0(0)	0(0)	2(50)
Other reasons (n=11), n (%)	3(27.27)	1(9.09)	2(18.18)	6(54.54)

## Discussion

In this single-center study we assessed factors influencing refusal of admission to ICU and the impact on mortality of being denied ICU admission. The main findings from this survey were the very high ICU refusal rate and the high mortality rate at 28 days, in particular, in patients considered “too sick to benefit”. Nevertheless, refusal due to unavailable ICU beds was a common occurrence. Higher mortality rate was shown among rejected patients due to unavailability of ICU beds. Factors independently associated to refusal to ICU admission were absence of acute respiratory failure and request by phone. In this study, refusal of ICU admission was defined as refusal at the first time; patients initially refused but admitted later were counted in the rejected patients' group. Indeed, the refusal rate (79.5%) was high as compared to the 73% [7], 38% [11] and 24% [6] rates reported in earlier studies. However, this rate was lower than that found in a French study where the rate was 88% [14]. This finding may be influenced by the fact that 128 of 260 patients (50%) confronted the problem of unavailability of beds in the ICU. Also, it is may be related to the inclusion of all patients for whom ICU admission was requested at the first time. The reasons for refusal given by ICU physicians confirmed previous findings [15, 16] although they differed in frequency. In this study, the most frequent reasons were unavailability of beds (50%) and the patients considered “too sick to benefit” (22%) while less frequently the reason was patients considered “too well to benefit” (13%). However, in recent studies, patient being “too well” or “too ill” were the most frequent reasons while lack of bed availability was less frequent [8]. We found that ICU refusal was influenced by both organizational factors and patient related factors. Absence of acute respiratory failure and evaluation over the phone were determinants of refusal. Among recent studies, a strong effect of bed availability [10] and older age [16-18]. On ICU refusal was found. One latest study carried out in 2016 by a Brazilian group reported that although the number of regulated beds is within the recommended range, an increase in beds of 122.0% is required to guarantee system stability and of 134.0% for a maximum waiting time of six hours [10]. On the other hand, recent studies focused on a specific age group. Pintado et al [8] reported that age older than 75 years was associated to ICU refusal, while Le Gueno et al [7] and Joynt et al [11] found that age younger than 65 years was associated to ICU admission. Nevertheless, an earlier study showed that an age older than 85 years was associated to ICU refusal. In our study, there was no significant association between age and ICU admission and refusal. In agreement with previous findings, the diagnosis group was associated to ICU refusal [11]. Furthermore, a French group reported that patients with acute respiratory failure were the most patients admitted to ICU and phone admission was associated to ICU refusal [1]. Several studies showed that dependency [5, 8] and presence of co-morbidity [15] predicted ICU refusal, while in our study, no significant association was found. In this study, the total mortality rate at 28 day in not admitted patients was 43.46%. An important mortality rate was observed in patients refused because being “too sick to benefit” (80.7%), unavailability of beds (26.56%) and less frequent patients being “too well to benefit” (5.71%). Our results confirmed previous evidence that patients being “too sick” revealed higher mortality rate [1, 7, 8, 13, 15]. However, these studies found that patients being “too well” had higher mortality rate than lack of bed availability. Nonetheless, a French study showed that mortality rate at 28 day of refused patients due to full unit was 30.1%, higher than 26.56% [5]. This survey concluded that delayed ICU admission due to a full unit at first referral is associated with increased mortality. This study had several limitations. First, it was conducted in a single center which may be let our results not applicable to other hospitals. Second, the lack of randomization may be a source of bias. Third, we did not measure the mortality rate in admitted patients to compare between the two groups. Our strengths are the period of the study of 6 months and 28 days follow-up. Thus, if we conducted a longer period, problems of patients' contact could be happen.

## Conclusion

In conclusion, our study of ICU admission requests suggests that refusal of admission to ICU was independently associated to one patient related factor (absence of acute respiratory failure) and one organizational factor (request by phone). The most frequent reasons for refusal were unavailability of beds, patients “too sick to benefit” and patients “too well to benefit”. Consequently, an important mortality rate was found especially in patients “too sick to benefit”. These results invite ICU physicians to discuss preferences about ICU admission and to improve the accuracy of data on ICU refusal rates and should evaluate their triage decisions and, if possible, routinely solicit patient preferences during medical emergencies, taking steps to ensure that ICU admission decisions are in line with the goals of the patient. Ultimately, these efforts will help ensure that scarce ICU resources are used most effectively and efficiently.

### What is known about this topic

- Among recent studies, a strong effect of bed availability and older age On ICU refusal was found.

### What this study adds

- Our study of ICU admission requests suggests that refusal of admission to ICU was independently associated with the absence of acute respiratory failure and request patients by phone without clinical evaluation.

## Competing interests

The authors declare no competing interest.
